# Functional Dissection of *C. elegans* bZip-Protein CEBP-1 Reveals Novel Structural Motifs Required for Axon Regeneration and Nuclear Import

**DOI:** 10.3389/fncel.2019.00348

**Published:** 2019-07-31

**Authors:** Rose Aria Malinow, Phoenix Ying, Thijs Koorman, Mike Boxem, Yishi Jin, Kyung Won Kim

**Affiliations:** ^1^Section of Neurobiology, Division of Biological Sciences, University of California, San Diego, La Jolla, CA, United States; ^2^Department of Biology, Utrecht University, Utrecht, Netherlands; ^3^Convergence Program of Material Science for Medicine and Pharmaceutics, Department of Life Science, Multidisciplinary Genome Institute, Hallym University, Chuncheon, South Korea

**Keywords:** IMA-3, importin, nuclear localization, Tribbles, NIPI-3, C/EBP, structural-function domain

## Abstract

The basic leucine-zipper (bZIP) domain transcription factors CCAAT/enhancer-binding proteins (C/EBP) have a variety of roles in cell proliferation, differentiation, and stress response. In the nervous system, several isoforms of C/EBP function in learning and memory, neuronal plasticity, neuroinflammation, and axon regeneration. We previously reported that the *Caenorhabditis elegans* C/EBP homolog, CEBP-1, is essential for axon regeneration. CEBP-1 consists of 319 amino acids, with its bZIP domain at the C-terminus and a long N-terminal fragment with no known protein motifs. Here, using forward genetic screening with targeted genome editing, we have identified a unique domain in the N-terminus that is critical for its *in vivo* function. Additionally, we characterized three nuclear localization signals (NLS) in CEBP-1 that act together to mediate CEBP-1’s nuclear import. Moreover, the Importin-α, IMA-3, can bind to CEBP-1 via one of the NLS. *ima-3* is ubiquitously expressed in all somatic cells, and *ima-3* null mutants are larval lethal. Using Cre-lox dependent neuron-specific deletion strategy, we show that *ima-3* is not critical for axon development, but is required for axon regeneration in adults. Together, these data advance our understanding of CEBP-1’s function, and suggest new regulators that remain to be identified to expand the CEBP-1 protein interactome.

## Introduction

CCAAT/enhancer-binding proteins (C/EBP) are conserved basic leucine-zipper (bZIP) domain transcription factors that are widely expressed and have a variety of roles in cell proliferation, differentiation, and stress response (Ramji and Foka, 2002; Yang et al., 2017). In neurons, C/EBPs have been linked to learning and memory (Alberini et al., 1994; Lee et al., 2012) as well as neuronal repair after injury (Nadeau et al., 2005; Yan et al., 2009; Aleksic and Feng, 2012; Lopez de Heredia and Magoulas, 2013). Expression and function of C/EBPs are regulated at multiple levels. For example, humans and mice have six C/EBP genes, CEBPα–ζ, and each C/EBP shows temporally regulated activity during development and in different tissue types (Ramji and Foka, 2002). While the bZIP domain of C/EBPs plays essential roles in DNA binding and transcriptional function, a variety of regulatory domains residing within the N- or C-terminus modulate the transcriptional activity and contribute to distinct functional outcomes.

Axon regeneration after nerve injury requires the activation of multiple pro-regenerative programs, including early calcium waves, activation of mitogen-activated protein kinase (MAPK) and signal transduction through various effectors (Ghosh-Roy et al., 2010; Shin et al., 2012; Cho et al., 2015; Mahar and Cavalli, 2018). To activate expression of injury-responsive genes, injury signaling must be transmitted retrogradely from axonal lesion sites to the soma and nucleus. The importin-dependent nucleocytoplasmic transport of transcription factors has been proposed as a common mechanism for linking axonal signaling to nuclear response (Hanz et al., 2003; Yudin et al., 2008; Perry et al., 2012). There are two types of importins in the classical nuclear import pathway, Importin-α and Importin-β: Importin-α directly binds to the nuclear localization signal (NLS), a recognition motif for nucleocytoplasmic transport factors at low affinity, whereas Importin-β binds to Importin-α and increases its affinity for NLS binding (Goldfarb et al., 2004; Mason et al., 2009). Several Importin-α isoforms are reported to be localized in the axons of rodents, such as sciatic nerves under both naïve and injury conditions (Hanz et al., 2003). Moreover, Importin β1 is induced only after injury in sensory axons of mice and rats, and forms a heterodimer with Importin-α (Perry et al., 2012).

*Caenorhabditis elegans* has three importin-α proteins (IMA-1/-2/-3) and three Importin-β proteins (IMB-1/-2/-3) (Geles and Adam, 2001; Geles et al., 2002). All Importin-α proteins share common sequence features, including an Importin β-binding (IBB) domain and ten tandem armadillo (ARM)-like repeats; all Importin-β proteins contains tandem repeats of HEAT domain (Goldfarb et al., 2004). Previous studies have focused on the roles of Importin-α proteins in the germline and early embryo development to regulate the mitotic cell cycle (Geles and Adam, 2001; Geles et al., 2002). The function of importins in the nervous system is largely unexplored.

In this paper, we investigate the regulation of CEBP-1, one of the *C. elegans* homolog of C/EBP transcription factors, which is essential for axon regeneration (Yan et al., 2009) and also functions in other cellular stress pathways (Bounoutas et al., 2011; Kim et al., 2016). CEBP-1 consists of a canonical bZIP domain at the C-terminus, with a long N-terminal region having no known protein motifs. Here, through forward genetic analyses, we have identified a stretch of 15 amino acids at the N-terminus of CEBP-1 that is critical for its *in vivo* function and axon regeneration. The region containing this unique domain has a predicted propensity to form alpha helices. We also dissected the role of three nuclear localization motifs in CEBP-1 for its nuclear import. We find that CEBP-1 interacts with IMA-3/Importin-α via one of the NLSs and that the nuclear transport of CEBP-1 is partially mediated by IMA-3. Importantly, we show that IMA-3 is critical for axon regeneration, supporting conserved roles of importin. These data reveal new insights into the molecular understanding of neuronal response to injury.

## Materials and Methods

### *C. elegans* Culture

Strains were maintained on NGM plates at 20°C as described previously (Brenner, 1974). Alleles and genotypes of strains are summarized in Table 1. We followed standard procedures to generate new transgenes. Plasmid and transgene information is in Table 2. Transgenes were introduced into mutants by genetic crossing or by microinjection, and genotypes for all mutations were confirmed by PCR or sequencing.

**TABLE 1:** Strains.

**Strain name**	**Genotype**
CZ24853	*nipi-3(ju1293); juEx7152[nipi-3(+); Pmyo-2::gfp; Phsp::peel-1]*
CZ25378	*cebp-1(ju1518) nipi-3(ju1293) X* (Figure 1A, #1)
CZ25379	*cebp-1(ju1519) nipi-3(ju1293) X* (Figure 1A, #2)
CZ25380	*cebp-1(ju1520) nipi-3(ju1293) X* (Figure 1A, #4)
CZ25381	*cebp-1(ju1521) nipi-3(ju1293) X* (Figure 1A, #5)
CZ26015	*cebp-1(ju1588) nipi-3(ju1293) X* (Figure 1A, #6)
CZ26014	*cebp-1(ju1587) nipi-3(ju1293) X* (Figure 1A, #7)
CZ26019	*cebp-1(ju1592) nipi-3(ju1293) X* (Figure 1A, #8)
CZ26017	*cebp-1(ju1590) nipi-3(ju1293) X* (Figure 1A, #9)
CZ27135	*cebp-1(ju1685) nipi-3(ju1293) X* (Figure 1A, #10)
CZ27136	*cebp-1(ju1686) nipi-3(ju1293) X* (Figure 1A, #11)
CZ26018	*cebp-1(ju1591) nipi-3(ju1293) X* (Figure 1A, #12)
CZ26013	*cebp-1(ju1586) nipi-3(ju1293) X* (Figure 1A, #13)
CZ26016	*cebp-1(ju1589) nipi-3(ju1293) X* (Figure 1A, #14)
CZ25382	*cebp-1(ju1522) nipi-3(ju1293) X*
CZ25383	*cebp-1(ju1523) nipi-3(ju1293) X*
CZ25384	*cebp-1(ju1524) nipi-3(ju1293) X*
CZ25385	*cebp-1(ju1525) nipi-3(ju1293) X*
CZ25386	*cebp-1(ju1526) nipi-3(ju1293) X*
CZ25387	*cebp-1(ju1527) nipi-3(ju1293) X*
CZ25388	*cebp-1(ju1528) nipi-3(ju1293) X*
CZ25389	*cebp-1(ju1529) nipi-3(ju1293) X*
CZ25390	*cebp-1(ju1530) nipi-3(ju1293) X*
CZ25391	*cebp-1(ju1531) nipi-3(ju1293) X*
CZ25392	*cebp-1(ju1532) nipi-3(ju1293) X*
CZ10969	*Pmec-7::gfp(muIs32) II*
CZ16489	*Pmec-7::gfp(muIs32) II; cebp-1(ju634) X* (Figure 1A, #3)
CZ27204	*Pmec-7::gfp(muIs32) II; cebp-1(ju1521) X* (Figure 1A, #5)
CZ27464	*Pmec-7::gfp(muIs32) II; cebp-1(ju1590) X* (Figure 1A, #9)
CZ17180	*Pcebp-1(2.2 kb)::cebp-1(aa 1–319)::gfp::cebp-1 3’UTR(juEx5097)*
CZ17181	*Pcebp-1(2.2 kb)::cebp-1(aa 1–319)::gfp::cebp-1 3’UTR(juEx5098)*
CZ17330	*Pcebp-1(2.2 kb)::gfp::cebp-1 3′UTR(juEx5094)*
CZ17371	*Pcebp-1(2.2 kb)::gfp::cebp-1 3′UTR(juEx5095)*
CZ21002	*cebp-1(tm2807) X; Pcebp-1::CEBP-1(aa 235–319)::gfp::cebp-1 3′UTR(juEx6336)*
CZ21003	*cebp-1(tm2807) X; Pcebp-1::CEBP-1(aa 235–319)::gfp::cebp-1 3′UTR(juEx6337)*
CZ19782	*Pcebp-1::cebp-1(aa 1–230)::gfp:cebp-1 3′UTR(juEx5995)*
CZ19783	*Pcebp-1::cebp-1(aa 1–230)::gfp:cebp-1 3′UTR(juEx5996)*
CZ20998	*cebp-1(tm2807) X; Pcebp-1::cebp-1(aa 1–175)::gfp::cebp-1 3′UTR(juEx6332)*
CZ20999	*cebp-1(tm2807) X; Pcebp-1::cebp-1(aa 1–175)::gfp::cebp-1 3′UTR(juEx6333)*
CZ21000	*cebp-1(tm2807) X; Pcebp-1::cebp-1(aa 1–160)::gfp::cebp-1 3′UTR(juEx6334)*
CZ21001	*cebp-1(tm2807) X; Pcebp-1::cebp-1(aa 1–160)::gfp::cebp-1 3′UTR(juEx6335)*
CZ20707	*cebp-1(tm2807) X; Pcebp-1::cebp-1(aa 1–115)::gfp::cebp-1 3′UTR(juEx6252)*
CZ20708	*cebp-1(tm2807) X; Pcebp-1::cebp-1(aa 1–115)::gfp::cebp-1 3′UTR(juEx6253)*
CZ18806	*Prgef-1::flag-cebp-1::gfp::cebp-1 3′UTR(juSi127) II*
CZ19790	*Prgef-1::flag-cebp-1-gfp::cebp-1 3’UTR(juSi127) II; ima-3(ok715) IV / nT1(qIs51) IV; V*
CZ22313	*ima-3(ok715) IV / nT1 IV; V; Pcebp-1::cebp-1N (aa 1–230)::gfp:cebp-1 3′UTR(juEx5995)*
CZ19324	*Prgef-1::flag::cebp-1(K162A, R164A, K168A, R169A)::gfp::cebp-1 3′UTR(juSi140) II*
CZ19784	*Pcebp-1::cebp-1(aa 1–230, K162A, R164A, K168A, R169A)::gfp:cebp-1 3′UTR(juEx5997)*
CZ19785	*Pcebp-1::cebp-1(aa 1–230, K162A, R164A, K168A, R169A)::gfp:cebp-1 3′UTR(juEx5998)*
CZ18767	*Pima-3::gfp::ima-3 3′ UTR(juEx5633)*
CZ18768	*Pima-3::gfp::ima-3 3′ UTR(juEx5634)*
CZ18435	*Pmec-7::gfp(muIs32) II; ima-3(tm1100)*
CZ10175	*Pmec-4::gfp(zdIs5) I*
CZ20408	*Pmec-4::gfp(zdIs5) I; Pima-3::lox2272::ima-3::lox2272::ima-3 3′UTR(juSi167) V; ima-3(ok715) IV*
CZ20407	*Pmec-4::gfp(zdIs5) I; Pima-3::ima-3::ima-3 3’UTR(juSi169) V; ima-3(ok715) IV; Pmec-7::nCre(juEx6042)*
CZ20406	*Pmec-4::gfp(zdIs5) I; Pima-3::lox2272::ima-3::lox2272::ima-3 3’UTR(juSi167) V; ima-3(ok715) IV; Pmec-7::nCre(juEx6042)*
CZ13799	*Punc-25::gfp(juIs76) II*
CZ21665	*Punc-25::gfp(juIs76) II; Pima-3::lox2272::ima-3::lox2272::ima-3 3’UTR(juSi167) V; Punc-25::nCRE(juEx6510)*
CZ21541	*Punc-25::gfp(juIs76) II; ima-3(ok715) IV; Pima-3::lox2272::ima-3::lox2272::ima-3 3’UTR(juSi167) V; Punc-25::nCRE(juEx6510)*
CZ21650	*Pflp-13::snb-1::gfp(juIs137) II; ima-3(ok715) IV / nT1 IV; V*
CZ18807	*Pmec-4::gfp(zdIs5) I; Prgef-1::flag::cebp-1::gfp (juSi127) II; cebp-1(tm2807) X*
CZ19326	*Pmec-4::gfp(zdIs5) I; Prgef-1::flag::cebp-1(K162A, R164A, K168A, R169A)::gfp (juSi140) II; cebp-1(tm2807) X*

**TABLE 2:** Plasmids and transgenes.

**Plasmid name**	**Description**	**Transgene**
pCZGY2507	*Pcebp-1::cebp-1(aa 1–319)::gfp::cebp-1 3′UTR*	*juEx5097, juEx5098*
pCZGY2505	*Pcebp-1::gfp::cebp-1 3′UTR*	*juEx5094, juEx5095*
pCZGY2550	*Pcebp-1::cebp-1(aa 235–319)::gfp::cebp-1 3′UTR*	*juEx6336, juEx6337*
pCZGY2506	*Pcebp-1::cebp-1(aa 1–230)::gfp:cebp-1 3′UTR*	*juEx5995, juEx5996*
pCZGY2548	*Pcebp-1::cebp-1(aa 1–175)::gfp::cebp-1 3′UTR*	*juEx6332, juEx6333*
pCZGY2549	*Pcebp-1::cebp-1(aa 1–160)::gfp::cebp-1 3′UTR*	*juEx6334, juEx6335*
pCZGY2546	*Pcebp-1::cebp-1(aa 1–115)::gfp::cebp-1 3′UTR*	*juEx6252, juEx6253*
pCZGY2524	*Prgef-1::flag::cebp-1(aa 1–319)::gfp::cebp-1 3′UTR*	*juSi127*
pCZGY3376	*Prgef-1::cebp-1(aa 1–319, K162A, R164A, K168A, R169A)::gfp::cebp-1 3′UTR*	*juSi140*
pCZGY3377	*Pcebp-1::cebp-1(aa 1–230, K162A, R164A, K168A, R169A)::gfp::cebp-1 3′UTR*	*juEx5997, juEx5998*
pCZGY2529	*ima-3 transcriptional reporter*	*juEx5633, juEx5634*
pCZGY2531	*ima-3 flanked by two lox2272 sites*	*juSi167*
pCZGY2532	*ima-3 gDNA*	*juSi169*
pCZGY1657	*Pmec-7::nCre*	*juEx6042*
pCZGY3225^#^	*Punc-25::nCre*	*juEx6510*
pMA122^##^	*Phsp::peel-1*	*juEx7152*
pCZGY1095	*Pmyo-2::gfp*	*juEx7152*
pCZGY3044	*nipi-3 gDNA*	*juEx7152*
pCZGY3083	AD-CEBP-1(aa 1–319) FL	NA
pCZGY2522	AD-CEBP-1(aa 1–235)	NA
pCZGY2520	AD-CEBP-1(aa 1–115)	NA
pCZGY3084	AD-CEBP-1(aa 117–235)	NA
pCZGY3100	AD-CEBP-1(aa 176–235)	NA
pCZGY3378	BD-CEBP-1(aa 237–319)	NA
pCZGY3081	BD-IMA-3(aa 1–514) FL	NA
pCZGY3082	BD-IMA-3(aa 278–514)	NA
pCZGY3085	AD-IMA-3(aa 1–514) FL	NA
pCZGY3373	AD-CEBP-1(aa 1–319, K162A, R164A, K168A, R169A)	NA
pCZGY3374	AD-CEBP-1(aa 1–319, K204A, K205A, K207A)	NA
pCZGY3375	AD-CEBP-1(aa 1–319, K239A, R240A)	NA

*^#^pCZGY3225 (Chen et al., 2015). ^##^pMA122 (Seidel et al., 2011).*

### Isolation of Novel *cebp-1* Mutations in a Suppressor Screen of *nipi-3(0)* Mutants

We previously reported that loss-of-function mutations of *cebp-1* suppress larval lethality of the Tribbles kinase *nipi-3(ju1293)* null mutant (Kim et al., 2016). We performed a large-scale suppressor screen using an efficient selection scheme. Briefly, we mutagenized L4 animals of the genotype: *nipi-3(ju1293); juEx7152[nipi-3(+); Pmyo-2::gfp; Phsp::peel-1]* using ethyl methane sulphonate (EMS), following standard procedures (Brenner, 1974). F2 progeny were subjected to heat shock at 37°C for 1 h, which induced the expression of the toxic protein PEEL-1 to kill any animals whose survival depended on the expression of *nipi-3(+)* from the *juEx7152* transgene. Those lived to fertile adults without *juEx7152* likely contained a mutation that suppressed *nipi-3(ju1293)* lethality. We then performed Sanger sequencing for *cebp-1* and identified mutations within *cebp-1* for 15 independent suppressor alleles (Table 1: *ju1518 – ju1532*).

### CRISPR/Cas9-Mediated Editing to Generate New Alleles Affecting the N’ Domain of CEBP-1

We generated missense, insertion and deletion alleles in the N’ domain of *cebp-1 (ju1586, ju1587, ju1588, ju1589, ju1590, ju1591, ju1592, ju1685, ju1686)* using the co-CRISPR method (Friedland et al., 2013). We designed one single guide RNA (sgRNA; 5′-GCAACGUGACCGCGAACGCC-3′) to target CCA for Glu61 in the N’ domain of the *cebp-1* gene. A mixture of *cebp-1* crRNA (0.3 μL of 200 μM), *dpy-10* crRNA (0.3 μL of 200 μM), tracrRNA (0.9 μL of 100 μM), Cas9 protein (3.5 μL of 40 μM) was injected into CZ24853 (*nipi-3(ju1293); juEx7152[nipi-3(+); Pmyo-2::gfp; Phsp::peel-1]*). F1 animals displaying dumpy and/or normal animal growth reaching adulthood without *juEx7152* transgene were propagated to the F2 generation. A total of 139 independent F2 isolates that were confirmed for suppression of *nipi-3(ju1293)* larval lethality were then analyzed by PCR and Sanger sequencing to identify changes in *cebp-1.* The animals containing large deletions based on size of PCR amplification products were not analyzed further, because they most likely altered protein translation affecting the downstream bZIP domain. We focused only on those having missense or small in-frame deletions and insertions within N’ domain.

### Yeast Two-Hybrid Screen and Assays

Yeast two-hybrid screens were performed as previously described (Boxem et al., 2008). We found that full-length and some fragments of CEBP-1 exhibited auto-activation activity in yeast two-hybrid assay. We used two partial CEBP-1 proteins, CEBP-1(aa 1–73) and CEBP-1(aa 117–235), as baits to screen a *C. elegans* library made of normalized complementary DNAs (cDNAs). No prey was identified with CEBP-1(aa 1–73) bait, whereas multiple clones of IMA-3 were found to interact with CEBP-1(aa 117–235).

We further verified the interaction in a different yeast two-hybrid assay system (Clontech, Mountain View, CA, United States). Full-length or fragments of cDNAs of CEBP-1 or IMA-3 were cloned into pACT2 (GAL4 DNA-binding domain) or pBTM116 (LexA DNA-binding domain) vectors. Pairs of plasmids were co-transformed into yeast strain L40, and selected on agar plates with synthetic defined (SD) minimal medium lacking leucine and tryptophan to obtain double transformants. A single clone was picked from each transformation and cultured until OD_600_ = 1. Yeast cells were then plated in a dilution series of 10–1000 times by pipetting 5 μl per spot onto SD medium plates lacking leucine, tryptophan and histidine containing 10 mM 3-Amino-1,2,4-triazole (3-AT). β-galactosidase assays were performed using the Thermo Scientific Yeast beta-galactosidase Assay kit as described by the manufacturer.

### Neuron-Specific Deletion of *ima-3* Using Cre-Lox System

We constructed pCZGY2531 and pCZGY2532 (Table 2) and generated a single-copy transgene containing either *ima-3* full-length genomic DNA flanked by two Lox2272 sites (*juSi167*) or *ima-3* genomic DNA (*juSi169*), respectively. Both transgenes fully rescued the larval lethality and adult sterility of *ima-3(ok715)* null animals. We used transgenes *juEx6042[Pmec-7::nCre]* (Chen et al., 2015) or *juEx6510[Punc-25::nCre]*, respectively to excise the floxed copy of *ima-3(+)* in mechanosensory neurons or GABAergic motor neurons, respectively (Table 1 for the strain information).

### Laser Axotomy and Axon Regeneration Analysis

We cut mechanosensory PLM axons using *muIs32[Pmec-7::gfp]* or *zdIs5[Pmec-4::gfp]* and GABAergic motor neuron commissures (VD3, DD2, and VD4) using *juIs76[Punc-25::gfp]* as previously described (Wu et al., 2007). Briefly, we anesthetized worms for surgery and imaging using 1% 1-phenoxy-2-propanol (TCI America, Portland, OR, United States) in M9 buffer and 0.1% in the agar pad. We mounted ∼10 L4 worms expressing GFP-labeled axons on agar pad with a cover slip. The neuronal axons were severed at about 40 μm from soma, using a femtosecond laser system (Verdi G from Coherent) and imaged using a Zeiss Axiovert 200M spinning disk confocal microscope with a Yokogawa CSU-XA1 head and a Photometrics Cascade II EMCCD camera controlled by μManager^1^. We recovered the worms immediately following axotomy, by removing the cover slip and then transferring them onto NGM plates seeded with OP50. Animals were cultured at 20°C for 24 h and remounted for confocal imaging using a Zeiss LSM510 or LSM710. We measured axon length using ImageJ (NIH) and subtracted the 24 h axon length from the 0 h axon length to assess the axon regrowth length.

### Quantification and Statistical Analysis

Statistical analysis was performed using GraphPad Prism 5. Significance was determined using unpaired *t* -tests for two samples, one-way ANOVA followed by Tukey multiple comparison tests for multiple samples. For two nominal variables, Fisher’s exact test (two-tailed) was used. *P* < 0.05 (^*^) was considered statistically significant. ^*^P < 0.05; ^∗∗^*P* < 0.01; ^∗∗∗^*P* < 0.001. Data are shown as mean ± SEM. “n” represents the number of animals and is shown in graphs.

## Results

### Identification of a Functional Domain in the N-Terminus of CEBP-1

**FIGURE 1:**
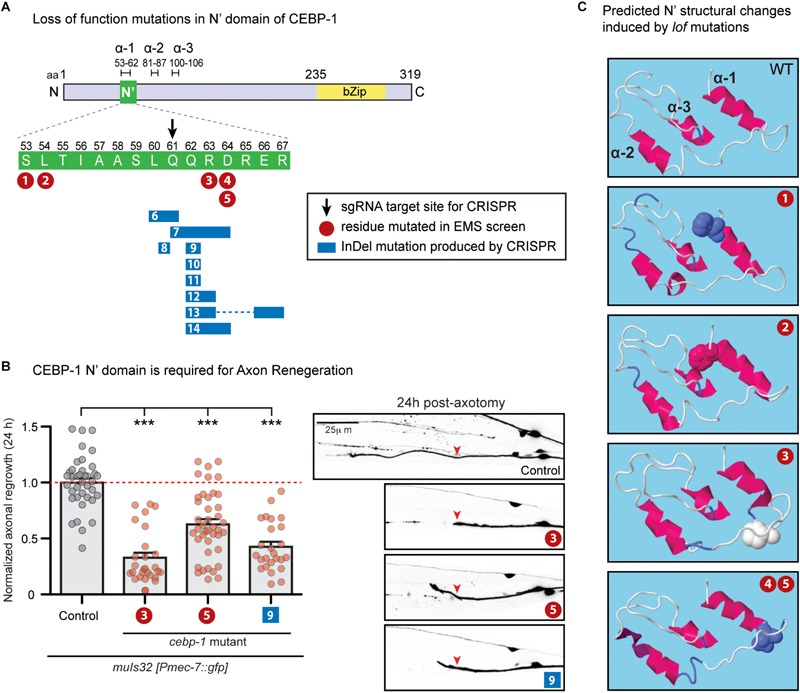
N’ functional domain in CEBP-1 is required for proper axon regeneration and protein structure formation. **(A)** CEBP-1 N’ domain mutants isolated in several forward genetic screens. Mutations 1–5 are missense mutations isolated in forward genetic screens utilizing EMS as a chemical mutagen. #1, *ju1518* S53F; #2, *ju1519* L54F; #3, *ju634* R63P; #4/5, *ju1520/ju1521* D64N. Mutations 6–14 were isolated in a forward genetic screen utilizing targeted CRISPR/Cas9 mutagenesis. The site targeted for double stranded DNA breakage is marked with a black arrow. These mutations do not cause a shift in frame, only add and/or delete amino acids adjacent to the cut site. #6, *ju1588Δ* (L60, Q61); #7, *ju1587*Δ(Q61, Q62, R63, D64) +HH; #8, *ju1592* L60[HSTRS]Q61; #9, *ju1590*ΔQ62; #10, *ju1685*ΔQ62 +HE; #11, *ju1686*ΔQ62 +HRG; #12, *ju1591*Δ(Q62, R63) +RPVTS; #13, *ju1586*Δ(Q62, R63)+H Δ(E66, R67); #14, *ju1589Δ* (Q62, R63, D64)+H. **(B)** PLM axon regrowth 24 h post-axotomy in *cebp-1(ju634)* mutants in PLM mechanosensory neurons. Length of regrowth was quantified by subtracting the initial length of the axon at 0 h from the length of the axon 24 h after injury. Data are shown as mean ± SEM. One-way ANOVA followed by Tukey’s multiple comparison test. ^∗∗∗^*p* < 0.001. Column 1: control, CZ10969 [*muIs32*]; Column 2: CZ21689 [*muIs32; cebp-1(ju634)*] (#3); Column 3: CZ27204 [*muIs32; cebp-1(ju1521)*] (#5); Column 4: CZ27464 [*muIs32; cebp-1(ju1590)*] (#9). Right: representative images of PLM axons 24 h post-axotomy. Red arrowhead, site of axotomy. **(C)** Alteration of predicted alpha helices structure upon *cebp-1* N’ mutations based on RaptorX. The S53F mutation (#1) makes a predicted break in α-2, producing two helices. The L54F mutation (#2) makes lengthening of α-1 and α-2, and the R63P mutation (#3) makes lengthening of α-2. The D64N mutation (#4/5) is predicted to cause the additional turn in the C’ end of α-2.

In previous genetic suppressor screens for loss-of-function mutations in *rpm-1*, a conserved E3 ubiquitin ligase, we isolated a missense mutation of *cebp-1, ju634*, that changes Arg63 to Pro in the N-terminus of CEBP-1 (Noma et al., 2014) (Figure 1A, #3). *cebp-1(ju634)* behaved as a genetic null, based on its effects to restore the synapse and axon developmental defects in *rpm-1* to normal (Noma et al., 2014). Moreover, in laser axotomy assay, we found that *cebp-1(ju634)* blocked injury-induced axon regrowth (Figure 1B), similarly to *cebp-1* null (Yan et al., 2009).

In a parallel investigation, we identified *cebp-1* to be negatively regulated by the Tribbles pseudokinase *nipi-3* in larval development (Kim et al., 2016). Loss of *nipi-3* results in larval lethality, which is fully suppressed by loss of *cebp-1.* We performed a large-scale genetic suppressor screen of *nipi-3(ju1293)* mediated developmental arrest (see Materials and Methods), and isolated four new missense mutations affecting amino acids adjacent to Arg63 (Figure 1A, #1,2,4/5). As all these point mutants phenocopied the *cebp-1(tm2807)* null, we hypothesized that this region may define a domain of functional significance.

To test this hypothesis, we designed genome-editing using a CRISPR/Cas9 directed mutagenesis with a sgRNA designed to target amino acid Glu61 (Figure 1A, black arrow). Following microinjection of sgRNA and Cas9 mixture into *nipi-3(ju1293)* mutants expressing a transgene, *juEx7152[nipi-3(+); myo-2p::gfp; Phsp::peel-1]*, we isolated >100 animals that were able to reach adulthood without the *juEx7152* rescue array (see Materials and Methods). Using PCR and Sanger sequencing, we then identified multiple alleles that contained missense mutations, as well as a single amino acid deletion or in-frame insertion of one or more amino acids (Figure 1A, #6–14). These new alleles all behaved similarly to each other and to *cebp-1* null, based on the suppression of *nipi-3(ju1293)* lethality. We also performed laser axotomy on two new mutations affecting residues adjacent to R63P. While *cebp-1(ju1590)* [#9: ΔQ62] reduced PLM axon regrowth to similar degree as *cebp-1(ju634)* [#3: R63P], *cebp-1(ju1521)* [#5: D64N] showed partially reduced axon regrowth, comparing to *cebp-1(ju634)* (Figure 1B). This finding is consistent with previous findings that axon regeneration is highly sensitive to CEBP-1 activity levels (Sharifnia et al., 2017), and CEBP-1(D64N) mutation causes partial loss of function. Together, these results strongly support that this stretch of 15 amino acids within the N-terminus of CEBP-1 defines a functional domain, named as N’ domain.

While this N’ domain is highly conserved among nematode homologs of *cebp-1*, we did not find homologous regions among C/EBPs in other species by BLAST search (Altschul et al., 1990). We next asked whether the N’ domain may form any secondary structure. Using RaptorX^2^ (an online predictor of protein structure) (Wang et al., 2016), we found that amino acids 55-106 in CEBP-1 N-terminus region has high propensity to form alpha helices (Figure 1C). Several missense mutations and amino acid insertion and deletion within the N’ domain are also predicted to alter the propensity of this region to form alpha helices (Figure 1C). Thus, this analysis suggests that the N’ region of CEBP-1 may form a highly structured domain to mediate interactions with other proteins.

### Investigating Domains of CEBP-1 Required for Its Nuclear Localization

**FIGURE 2:**
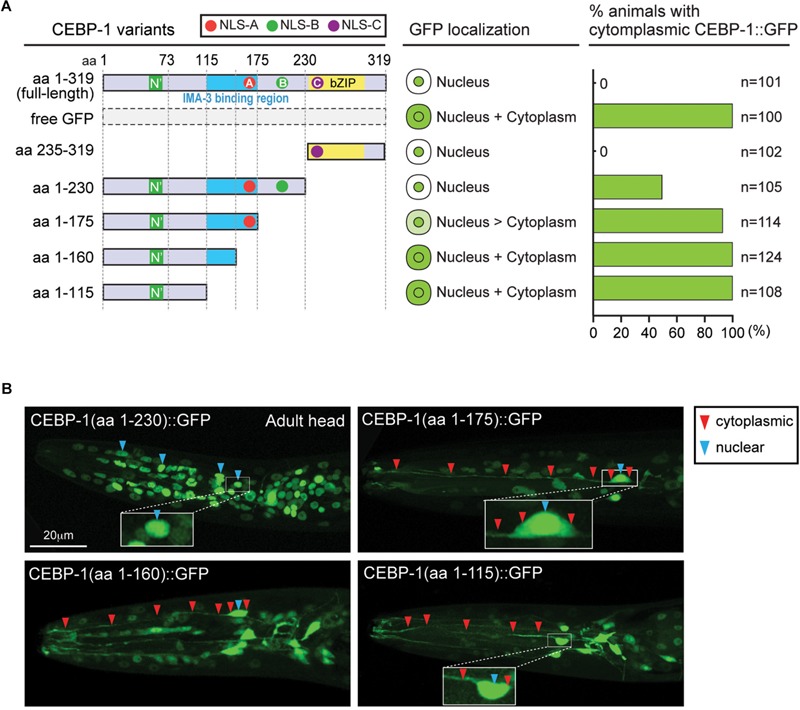
CEBP-1 is localized to the nucleus. **(A)** Subcellular localization of CEBP-1 variants fused to GFP in *C. elegans.***(B)** Representative confocal images of head parts of adult animals expressing CEBP-1 variants fused to GFP.

Full-length CEBP-1 is localized to the nucleus (Figure 2A). To gain clues to the function of N-terminal region of CEBP-1, we asked how the N-terminus of CEBP-1 may affect the nuclear localization of CEBP-1. We constructed a series of truncated CEBP-1 proteins tagged with GFP, and expressed them as transgenes under the *cebp-1* promoter. We observed that the C-terminal aa 235–319, including the bZIP domain, was localized to the nucleus, resembling the full-length CEBP-1 (Figure 2A), consistent with the general notion that bZIP domain has an inherent ability to localize to the nucleus. CEBP-1(aa 1-230) tagged with GFP was also largely localized in the nucleus (Kim et al., 2016). However, GFP fused in-frame to the N-terminal fragments of CEBP-1, CEBP-1(aa 1–115), and CEBP-1(aa 1–160), which include the N’ domain, showed no differential subcellular localization, resembling free GFP, while CEBP-1(aa 1–175) showed a partial nuclear localization (Figures 2A,B). Thus, the nuclear localization of CEBP-1 is gradually increased when progressively larger segments of the N-terminus of CEBP-1 is included. This analysis also suggests that the N’ domain of CEBP-1 may not play a strong role in mediating CEBP-1 nuclear localization.

### IMA-3 Can Bind the CEBP-1 N-Terminus

**FIGURE 3:**
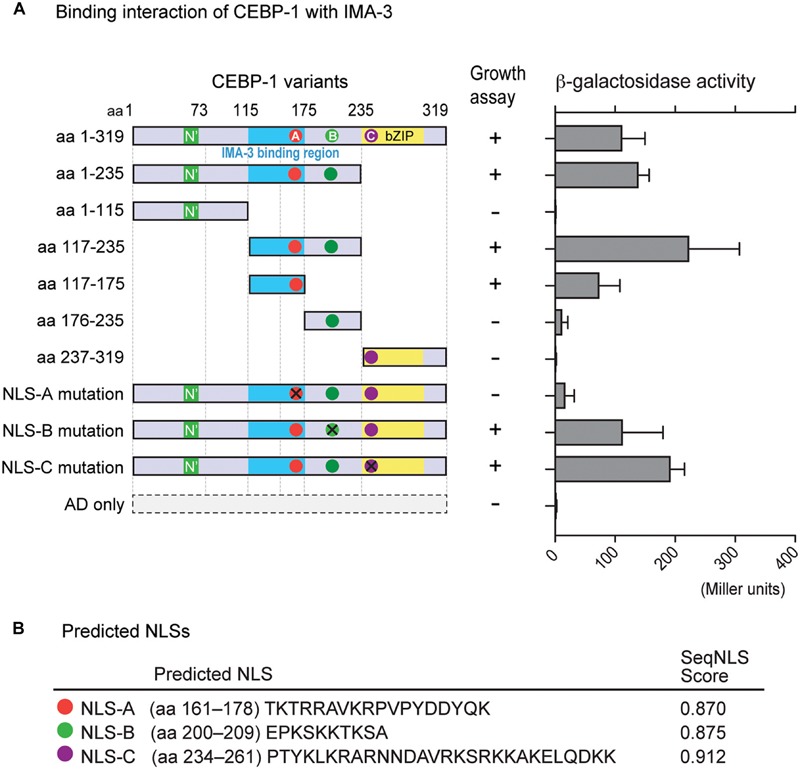
CEBP-1 interacts with IMA-3. **(A)** Delineation of the minimal CEBP region required for interaction with IMA-3. The indicated AD-CEBP-1 fragments and NLS mutants were tested for interaction with BD-IMA-3(aa 278–514), except the interaction test between BD-CEBP-1(aa 237–319) and AD-IMA-3(aa 1–514). **(B)** Nuclear localization signals (NLSs) of CEBP-1 predicted using the seqNLS program.

We next searched for CEBP-1 binding proteins by performing yeast two-hybrid screening. As the full-length CEBP-1 showed auto-activation activity in yeast two-hybrid assay, we carried out the screen using CEBP-1(aa 1–75) and CEBP-1(aa 117–235) as baits. CEBP-1(aa 1–75) bait did not yield any interactions, while multiple clones of IMA-3, the *C. elegans* importin-α, were isolated using CEBP-1(aa 117–235) as bait. We then verified the interaction using a different yeast two-hybrid assay system, and further narrowed the IMA-3 binding region to a stretch of 59 amino acid residues (aa 117–175) of CEBP-1 (Figure 3A).

As Importin-α is known to directly bind to the NLS, a recognition motif for nucleocytoplasmic transport factors (Goldfarb et al., 2004), we searched for potential NLSs in CEBP-1 using SeqNLS^3^ (Lin and Hu, 2013). This search revealed three potential NLSs within CEBP-1: TKTRRAVKRPVPYDDYQK (aa 161–178; NLS-A), EPKSKKTKSA (aa 200–209; NLS-B), and PTYKLKRARNNDAVRKSRKKAKELQDKK (aa 234–261; NLS-C) (Figure 3B). As NLS-A is localized within the region of CEBP-1 that is necessary for IMA-3 binding in the yeast two-hybrid assay, we further generated four point mutations changing the basic amino acids to Ala (K162A, R164A, K168A, and R169A). We found that such mutations abolished the interaction of CEBP-1 with IMA-3 in yeast (Figure 3A). Similar mutations in either NLS-B (K204A, K205A, K207A) or NLS-C (K239A, R240A) did not abolish the interaction of CEBP-1 with IMA-3 (Figure 3A). Therefore, we conclude that IMA-3 binds CEBP-1 via the NLS-A.

### *ima-3* Is Broadly Expressed in Somatic Cells Including Neurons

**FIGURE 4:**
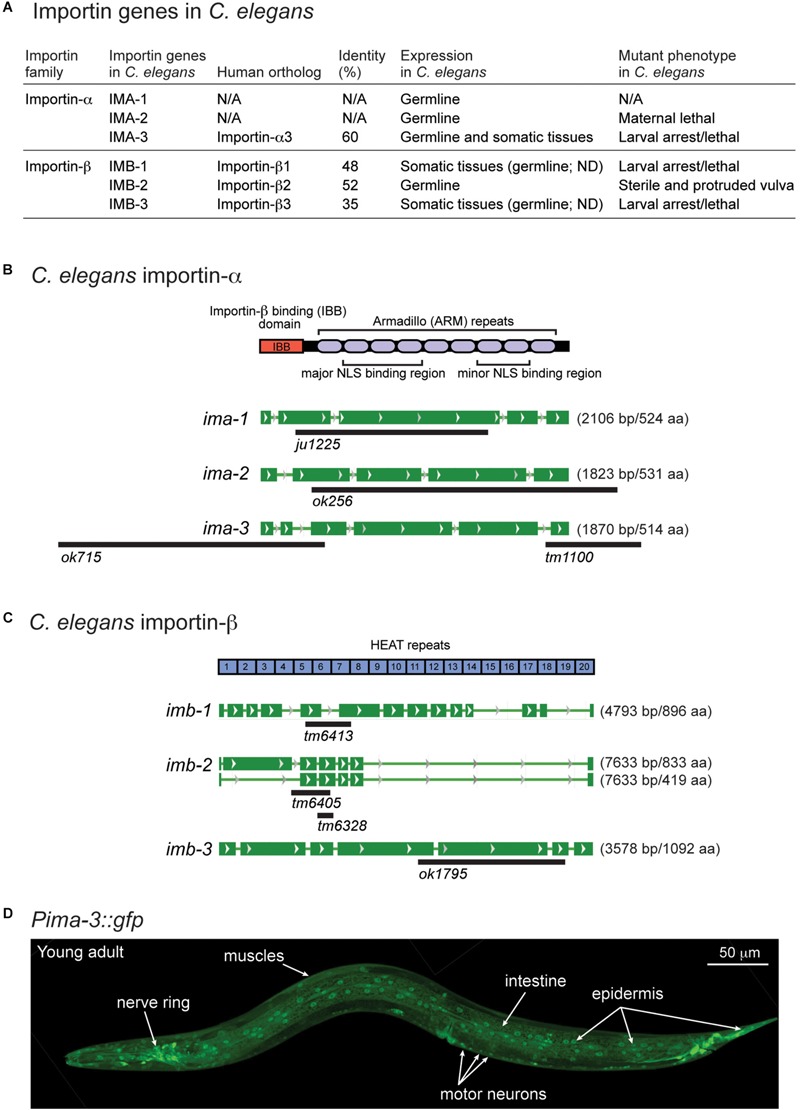
*ima-3* is expressed in many somatic tissues. **(A)** A summary table of *C. elegans* importins. **(B)** *C. elegans* importin-α genes and available mutations. **(C)** *C. elegans* importin-β genes and available mutations. **(D)** A representative confocal image of *C. elegans* importin-α3/*ima-3* expression (*Pima-3::gfp)* in a young adult animal.

There are two types of importins, α and β. Importin-α recognizes proteins with NLSs (cargo) and forms cargo/importin-α/importin-β trimetric complexes (Miyamoto et al., 2016). Like other organisms, *C. elegans* expresses both importin-α and -β genes (Figures 4A–C), including conserved (conventional) and species-specific (non-conventional) importin-α genes (Goldfarb et al., 2004). IMA-1 and IMA-2 are non-conventional importin-α proteins and IMA-3 is the only conventional importin-α protein that shares 60% identity of amino acid sequence with a human importin-α3 subtype (Figures 4A,B). Previous reports based on Northern blot analysis suggest that both *ima-1* and *ima-2* are expressed specifically in the germline, while *ima-3* is ubiquitously expressed in both germline and somatic tissues (Geles and Adam, 2001; Geles et al., 2002). Here, we determined the tissue expression pattern by generating transgenic GFP reporters driven by the 1.2 kb of 5′-upstream of *ima-3* (*Pima-3::gfp*). We observed GFP expression in most neurons and other somatic cell types including pharynx, intestine, epidermis, and muscles (Figure 4D). Thus, IMA-3 is broadly expressed in nearly all somatic tissues, and is likely the sole importin-α protein expressed in the neurons.

The *C. elegans* genome also encodes three importin-β genes (*imb-1, imb-2*, and *imb-3*), identified as orthologs of human importin-β genes (Figures 4A,C). All three Importin β amino acid sequences contain multiple HEAT repeats, which are found in many proteins involved in intracellular transport processes. Among three importin-β genes, null mutants for *imb-1* and *imb-3* display severe somatic defects and are arrested as larvae, suggesting their function in somatic cells, while *imb-2* appears to largely function in the germline as its null mutants are sterile adults.

### CEBP-1 Nuclear Localization Partly Depends on IMA-3

**FIGURE 5:**
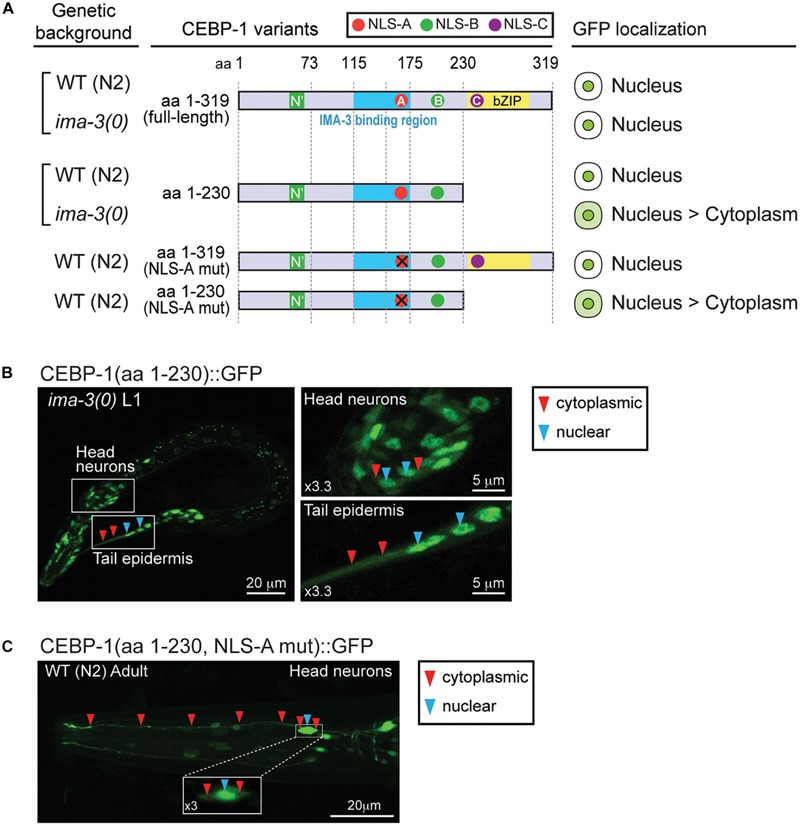
IMA-3 is partly required for CEBP-1 nuclear localization. **(A)** Subcellular localization of CEBP-1 variants fused to GFP in either wild type (WT) animals or *ima-3(ok715)* null mutants. **(B)** Representative image of whole L1 animal of *ima-3(ok715)* mutants expressing CEBP-1(aa 1–230)::GFP in the *ima-3(ok715)* mutant; left panels, enlarged images showing both nuclear and cytoplasmic expression of CEBP-1. **(C)** Representative image of a head part of adult animals expressing CEBP-1(aa 1–230, NLS-A mutation)::GFP in the WT animal; inset: head neuron cell body showing both nuclear and cytoplasmic expression of CEBP-1.

The identification of IMA-3 as a CEBP-1 binding protein raised the possibility that IMA-3 may regulate nuclear-cytoplasmic localization of CEBP-1. As CEBP-1(aa 1–175) that contains NLS-A is partially localized to the nucleus and CEBP-1(aa 1–230) that contains both NLS-A and NLS-B is predominantly localized to the nucleus, we reasoned that NLS-A and NLS-B may act redundantly to mediate cytoplasmic-nuclear import of CEBP-1. We examined how the subcellular expression of CEBP-1 was dependent on *ima-3* by crossing the GFP-tagged CEBP-1 transgenes into *ima-3(ok715)* mutants. Although the expression of the full-length CEBP-1::GFP in *ima-3(ok715)* was similar to that in wild type, we found that the nuclear expression of CEBP-1(aa 1–230) was reduced in *ima-3(ok715)* mutant animals (Figures 5A,B). Moreover, CEBP-1(aa1–230) that also contained the mutant NLS-A (K162A, R164A, K168A, and R169A), which abolished the interaction of CEBP-1 with IMA-3, showed increased cytoplasmic expression (Figures 5A,C). However, the nuclear localization of the full-length CEBP-1 with mutant NLS-A was unaltered, indicating NLS-C within bZIP domain has a dominant role in CEBP-1 nuclear localization. Together, these results support a conclusion that IMA-3 is involved in the cytoplasmic-nuclear import of CEBP-1, and also suggest that there are importin-independent pathways that ensure CEBP-1 nuclear localization.

### IMA-3 Is Required for Adult Axon Regeneration

**FIGURE 6:**
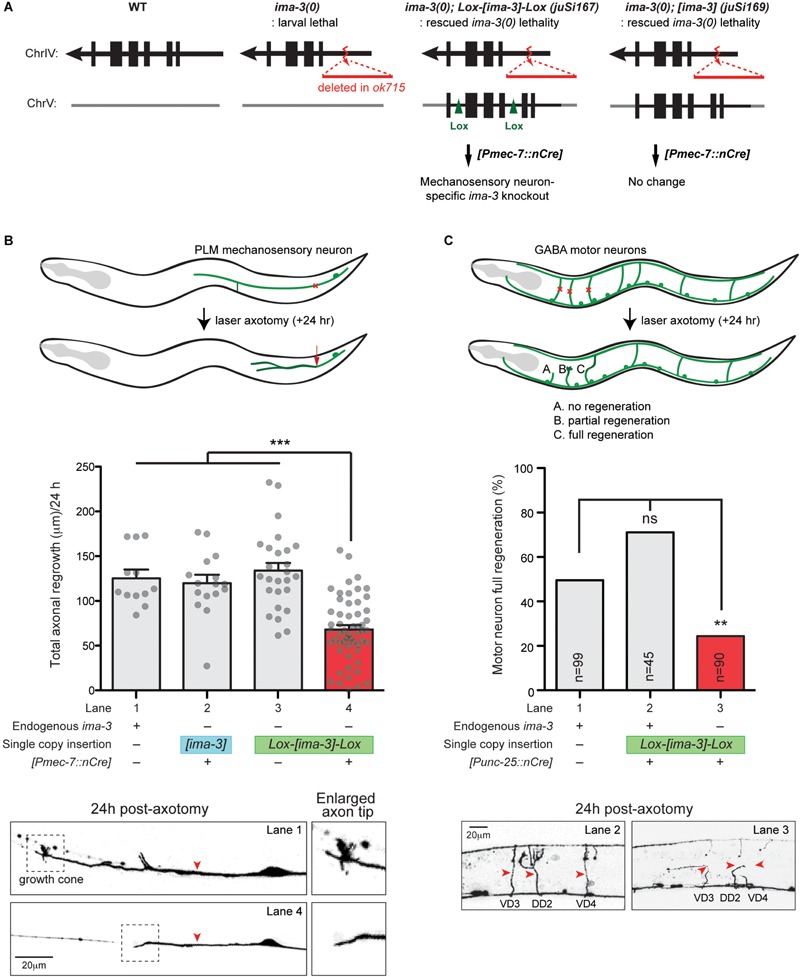
*ima-3* is required cell autonomously for PLM axon regeneration. **(A)** Schematic illustration of a strategy to generate *ima-3* mutation in mechanosensory neurons. The lethality of *ima-3(ok715)* is rescued by *juSi167[Lox2272-flanked ima-3 gDNA]* or *juSi169[ima-3 gDNA]*. Both *juSi167* and *juSi169* were crossed to *Pmec-7::nCre*, which deletes transgenic IMA-3 in mechanosensory neurons in *juSi167*, but not *juSi169* animals. **(B)** PLM axon regrowth 24 h post-axotomy in *ima-3(ok715)* mutation in mechanosensory neurons. One-way ANOVA followed by Tukey’s multiple comparison test. Data are shown as mean ± SEM. ^∗∗∗^*p* < 0.001. Bottom: representative images of PLM axons 24 h post-axotomy. Red arrowhead, site of axotomy. **(C)** GABAergic motor neuron full regeneration 24 h post-axotomy in *ima-3(ok715)* mutation in GABAergic motor neurons. Fisher’s exact test, two-tailed. Data are shown as mean. n, number of animals shown within columns. ns, not significant; ^∗∗^*p* < 0.01. Bottom: representative images of motor neurons (VD3, DD2, VD4) 24 h post-axotomy. Red arrowhead, site of axotomy.

Importin-dependent retrograde transport has been shown to play an important role to relay nerve injury signaling to promote axon regeneration in mammals (Hanz et al., 2003; Perry et al., 2012). To test whether importins are important in axon regeneration in *C. elegans*, we next investigated whether IMA-3 is required for axon regeneration in *C. elegans*. We first examined *ima-3(tm1100)* that contains a deletion at the 3′ end of the gene, removing last 35 aa and 3′ UTR (Figure 4B). *ima-3(tm1100)* homozygous animals are viable and grow similar to wild type (Geles and Adam, 2001). PLM axons developed normally, and also regenerated normally in *ima-3(tm1100)* (compared to wild type, 0.98 ± 0.06 normalized regrowth, *p* -value = 0.55, *n* = 17). The *ima-3(ok715)* null mutation removes the promoter and 5′ coding sequence, and the animals are larval lethal. We found that the mechanosensory neurons are born and developed normally in *ima-3(ok715)* larvae. To address effects of *ima-3* specifically in adult axon regeneration, we utilized a Cre-lox recombination system to examine neuron-specific deletion of *ima-3*. We generated two single-copy transgenes inserted at a defined location on chromosome V using the universal mosSCI technique (Frøkjær-Jensen et al., 2008). One consists of 4.3 kb *ima-3* genomic DNA (*juSi169; [ima-3]*), and another one with the same *ima-3* genomic DNA flanked by Lox2272 site (*juSi167; Lox-[ima-3]-Lox*) (Figure 6A). Both transgenes fully rescued the *ima-3(ok715)* lethality and adult sterility. We then introduced cell-specific transgenes expressing nuclear Cre recombinase (nCre) in mechanosensory neurons to eliminate IMA-3 production only in mechanosensory neurons (Figure 6A; *[Pmec-7::nCre]*). These animals showed no detectable growth or behavioral abnormality, and PLM axons were morphologically normal. In laser axotomy assays, we found that PLM axons in animals expressing unfloxed *ima-3(+)* and nCre displayed normal regrowth (Figure 6B, lane 2), whereas in animals containing floxed *ima-3*, expression of nCre resulted in severely impaired axon regrowth of PLM (Figure 6B, lane 4). We also found that while 30.7% of control (*zdIs5*) axons showed growth cone-like structure (*n* = 26) 24 h after axotomy, only 1.7% of the *ima-3* neuronal knockout animals had growth cone-like tips (*n* = 57) (Figure 6B, bottom panels), suggesting that *ima-3* likely affects the growth cone formation after injury. Similarly, we used another transgene expressing nCre in GABAergic motor neurons, and found that *ima-3* is also required for motor neuron axon regeneration (Figure 6C). In both Cre-mediated neuronal deletion of *ima-3* animals, the axon morphology of the mechanosensory neurons or GABAergic motor neurons was normal. Thus, we conclude that *ima-3* is dispensable for axon development, but is required for axon regeneration in both mechanosensory and motor neurons. To address whether the interaction between IMA-3 and CEBP-1 is necessary for PLM axon regeneration, we expressed a NLS-A mutant form of CEBP-1 (K162A, R164A, K168A, and R169A) in *cebp-1(0)*, and found that it rescued regeneration failure of PLM axons, similar to the expression of a wild type form of CEBP-1 [Total axonal regrowth length/24 h: CEBP-1(WT) = 97.99 ± 9.703 μm, *n* = 18; CEBP-1(NLS-A mutant) = 84.43 ± 10.48 μm, *n* = 15; not significant by unpaired Student’s *t* -test]. These results suggest that the interaction between CEBP-1 and IMA-3 via NLS-A does not play significant role in PLM axon regeneration.

## Discussion

### A Novel Functional Domain in CEBP-1

While previous work on CEBP-1 has shown that it is essential for axon regeneration, the only defined functional domain has been the highly conserved bZIP domain (Yan et al., 2009). In this study, through forward genetic screenings and a site-directed mutagenesis screening, we have identified a unique functional domain in the N’ terminus of CEBP-1. We showed that this domain is required for the function of CEBP-1 in the NIPI-3-mediated development pathway as well as the function in adult axon regeneration. Furthermore, analysis from protein structural and modeling prediction suggests that this domain resides within a highly structured region, and that this structure can be altered by the mutations isolated in our screens. A majority of CEBP homologues in other species contain regulatory domains in their N’ terminus that contribute to protein function (Hunter and Karin, 1992; Tsukada et al., 2011). For example, the transactivating domains in the N’ terminus of mouse CEBPα can bind directly to cyclin-dependent kinases and a chromatin remodeling complex, and mediate specific functional outcomes in cell cycle progression and epigenetic regulation (Nerlov, 2007). Thus, we propose that this newly identified domain in CEBP-1 likely impacts the transcriptional activity of CEBP-1 via binding to other unidentified factors. The interaction that involves this N’ domain likely leads to transcriptional activation as mutations in this domain phenocopy null mutations of CEBP-1. Further, it is worthy commenting on the CRISPR/Cas9-mediating genome editing technology. The sgRNA we designed showed high efficiency to guide Cas9 to the PAM sequence, as we observed all editing occurred 3′ downstream from the PAM site. As we were able to obtain a large number of editing mutants because of the efficient functional assay for *cebp-1* loss of function, our findings reveal a surprising degree of imprecise repair, ranging from a few nucleotide modification, insertion, deletion, to large deletion of several hundred nucleotides. Thus, this analysis raises caution for medical intervention, and urges deep studies of genome editing technology.

### CEBP-1 Nuclear Localization Depends on Multiple NLS Sites and Partly on IMA-3

In response to axon injury, there is a transient influx of calcium (Ghosh-Roy et al., 2010), leading to the activation of distinct pathways, some activating downstream transcription factors (Hammarlund and Jin, 2014; Hisamoto and Matsumoto, 2017). CEBP-1 is activated through the MAPKKK DLK-1 and PMK-3/p38 pathway (Yan et al., 2009). Another transcription factor required for axon regeneration, ETS-4, is activated by phosphorylation through a cAMP pathway (Li et al., 2015). These two transcription factors form a complex in the nucleus and promote the transcription of a transmembrane receptor SVH-2, which senses extracellular growth factors, leading to axon growth (Li et al., 2015). Although many genetic players in these pathways have been identified, the process of retrograde signaling is poorly understood.

Importin-mediated cytoplasmic-nuclear shuttling is known to broadly regulate retrograde signaling (Rishal and Fainzilber, 2014). In this study, we have identified at least three candidate nuclear localization sites on the CEBP-1 protein. The NLS-C motif within the bZIP domain appears to act dominantly and is sufficient for the localization of CEBP-1 to the nucleus. The other two NLS sites, NLS-A and NLS-B, can act together to facilitate the nuclear import of CEBP-1.

Our data also show that CEBP-1 interacts with IMA-3 via a stretch of 59 amino acid residues (aa 117–175) overlapping with NLS-A. We also showed that the expression of partial CEBP-1(aa 1–230) either containing mutations on the NLS-A or in the *ima-3(0)* mutant background was altered to be partially cytoplasmic. These results suggest that the expression pattern of CEBP-1 is at least partially dependent on IMA-3. In normal circumstances, full-length CEBP-1 protein does not seem to require Importin-α/IMA-3 for nuclear import, likely due to the dominant effects of NLS-C and DNA binding activity of bZip domain. A recent study suggests that three-dimensional context rather than NLS sequence determines Importin-α specificity for binding its partner protein (Sankhala et al., 2017). It is possible that Importin-α binding to CEBP-1 may be influenced by other regions of the protein in addition to the predicted NLSs. IMA-3 may facilitate the nuclear import of CEBP-1 under neuronal injury, where rapid action of CEBP-1 is necessary to activate cellular response. While we attempted to test this possibility, the low level of cytoplasmic pool of CEBP-1 precluded visible detection. It would be of future interest to define the extent and context of IMA-3-mediated CEBP-1 nuclear import.

### IMA-3/Importin-α3 Is Required for Adult Axon Regeneration

*C. elegans* IMA-3 is broadly expressed and likely functions in nearly all somatic tissues including neurons, like its homologs in other species. Thus, null mutations of *ima-3* and other importin genes display overall defects in organismal growth. In the arrested larvae, many neurons we examined showed normal axon morphology, and synapses in some motor neurons were also normal (Table 1), suggesting that importins are not essential for axon development, although it remains possible that axon development may be supported by maternally supplied importins. Using a neuron-specific deletion strategy to remove *ima-3* in post-mitotic neurons, we find no defects in axon development, indicating that axon growth and maintenance in larvae and adults likely does not rely on importins. In contrast, we show that neuronal depletion of IMA-3 causes significant reduction of axon regeneration following injury. We also find that IMA-3’s role in axon regeneration is likely independent of the nuclear import of CEBP-1. To our knowledge, this is the first demonstration of importin’s role in *C. elegans* neurons. This finding is also consistent with those studies from vertebrate axon injury studies. In rats, importin is required for the repair after nerve injury likely via retrograde transport of injury signaling molecules from injury site to the cell body (Hanz et al., 2003; Perry et al., 2012). As CEBP-1’s nuclear import tends to be also facilitated by IMA-3-independent pathway, an important challenge for the future is to identify the importin-independent retrograde transport mechanisms that are critical for promoting axon regeneration.

## Data Availability

All datasets generated for this study are included in the manuscript and/or the supplementary files.

## Ethics Statement

We primarily used *C. elegans* and *S. cerevisiae* as research organisms, which do not require animal protocols.

## Author Contributions

RM, YJ, and KK designed the work and drafted the manuscript. RM, PY, TK, MB, YJ, and KK performed the experiments and analyzed the data. RM, PY, MB, YJ, and KK reviewed and edited the manuscript. All authors have approved the final version of the manuscript and have agreed to be accountable for all aspects of the work regarding questions related to the accuracy or integrity of any part of the work.

## Conflict of Interest Statement

The authors declare that the research was conducted in the absence of any commercial or financial relationships that could be construed as a potential conflict of interest.

## Footnotes

1 www.micro-manager.org

2 http://raptorx.uchicago.edu

3 http://mleg.cse.sc.edu/seqNLS/
